# Lack of NWC protein (c11orf74 homolog) in murine spermatogenesis results in reduced sperm competitiveness and impaired ability to fertilize egg cells *in vitro*

**DOI:** 10.1371/journal.pone.0208649

**Published:** 2018-12-06

**Authors:** Michal Majkowski, Agnieszka Laszkiewicz, Lukasz Sniezewski, Pawel Grzmil, Bernadetta Pawlicka, Igor Tomczyk, Martyna Michniewicz, Violetta Kapusniak, Sylwia Janik, Grzegorz Chodaczek, Malgorzata Cebrat

**Affiliations:** 1 Laboratory of Molecular and Cellular Immunology, Hirszfeld Institute of Immunology and Experimental Therapy, Polish Academy of Sciences, Wroclaw, Poland; 2 Department of Genetics and Evolution, Institute of Zoology and Biomedical Research, Jagiellonian University, Krakow, Poland; 3 Vivarium, Hirszfeld Institute of Immunology and Experimental Therapy, Polish Academy of Sciences, Wroclaw, Poland; 4 Chair of Biostructure and Physiology, Department of Histology and Embryology, Wroclaw University of Environmental and Life Sciences, Wroclaw, Poland; 5 Laboratory of Confocal Microscopy, PORT Polish Centre for Technology Development, Wroclaw, Poland; University of Hyderabad, INDIA

## Abstract

NWC is an uncharacterised protein containing three strongly conserved domains not found in any other known protein. Previously, we reported that the NWC protein is detected in cells in the germinal layer in murine testes (strain: C57BL/6), and its knockout results in no obvious phenotype. We determined the NWC expression pattern during spermatogenesis, and found this protein in spermatocytes and round spermatids, but not in epididymal sperm. Although NWC knockout males are fertile, we further characterised their reproductive potential employing non-standard mating that better simulates the natural conditions by including sperm competition. Such an approach revealed that the sperm of knockout males fail to successfully compete with control sperm. After analysing selected characteristics of the male reproductive system, we found that *NWC* knockout sperm had a reduced ability to fertilize cumulus-intact eggs during IVF. This is the first report describing a subtle phenotype of *NWC* knockout mice that could be detected under non-standard mating conditions. Our results indicate that NWC plays an important role in spermatogenesis and its deficiency results in the production of functionally impaired sperm.

## Introduction

Spermatogenesis is a complex process involving the expression of a large number of genes [1–2]. The function of many of these genes remains unknown, resulting in an incomplete understanding of this process, sperm function and the causes of male infertility [3]. A standard approach to elucidating a gene function is to generate knockout (KO) mice and analyse their phenotypes. This strategy has helped to characterise hundreds of genes that are critical for the proper functioning of the male reproduction system ([4–5]). Finally, such research aims to better understand human reproduction and reasons of infertility. However, the approach has significant drawbacks. In particular, if a generated KO male is found to be fertile, the gene in question is classified as ‘not essential’ for male fertility. One must keep in mind, however, that fertility is most commonly examined under normal laboratory mating conditions, in which a male is housed in a single cage with one or two females, and the number of pups is taken as a fertility indicator [3]. The standard laboratory conditions do not reflect the natural environment, where mice are under the pressure of many other selective forces, including those affecting the function of the male reproduction system. Among the latter, sperm competition is of great importance. It takes place in the female reproductive track after promiscuous copulation with at least two males, whose sperm compete for the fertilization of available ova [6]. Therefore, analysis of gene function in the male reproductive system may require a detailed examination of fertility parameters that take into account complex mating behaviour. Such attempts may reveal subtle or complex reproductive phenotypes and, eventually, the role of a given gene/protein in a cell and in a particular process [7–9].

*NWC* (*B230118H07Rik*; homolog of human uncharacterised *c11orf74* gene) is the third evolutionarily conserved gene identified within the recombination activating gene (RAG) locus [10]. *NWC* is transcribed in all tissues except mature lymphocytes [11–14]. However, the presence of the NWC protein in mice has been detected exclusively in the germinal layer in the testes [15]. The predicted structure of the NWC protein revealed three domains strongly conserved among vertebrates, two of which are also conserved in invertebrate species [16]. Interestingly, all three domains share no similarity to any other proteins described in available databases. Although NWC is highly evolutionarily conserved, its cellular function remains unknown. The NWC-knockout (NWC-KOMPcre in [15]) mice revealed no apparent phenotype and are fertile in standard laboratory conditions. A histochemical analysis of testicular sections of these mice revealed the preservation of a layer of cells in the seminiferous tubules, and immunoprecipitation experiments showed that NWC interacts with proteins mostly involved in retrograde ciliary transport: IFT-139, IFT-144, IFT-122, IFT-43 and dynein light chain roadblock-type 1 [15].

In the present study, we examine NWC-KO males in terms of possible defects of male reproduction. We determined the NWC localisation and expression pattern during spermatogenesis. Subsequently, by employing non-standard mating that lead to sperm competition, we found that the sperm of knockout males fail to successfully compete with control sperm. Furthermore, we analysed selected characteristics of the male reproductive system and found NWC knockout results in: (1) an increased proportion of sperm with an abnormal head morphology and (2) their reduced ability to undergo acrosome reaction when stimulated. Finally, employing *in vitro* fertilization, we revealed that the sperm derived from NWC knockout had a reduced ability to fertilize cumulus-intact eggs. This is the first report describing a significant phenotype of the murine NWC knockout model. Our results indicate that NWC plays an important role in spermatogenesis and its loss in this process results in the production of functionally impaired sperm.

## Results

### Localisation of NWC protein in the testes of adult mice

**Fig 1:**
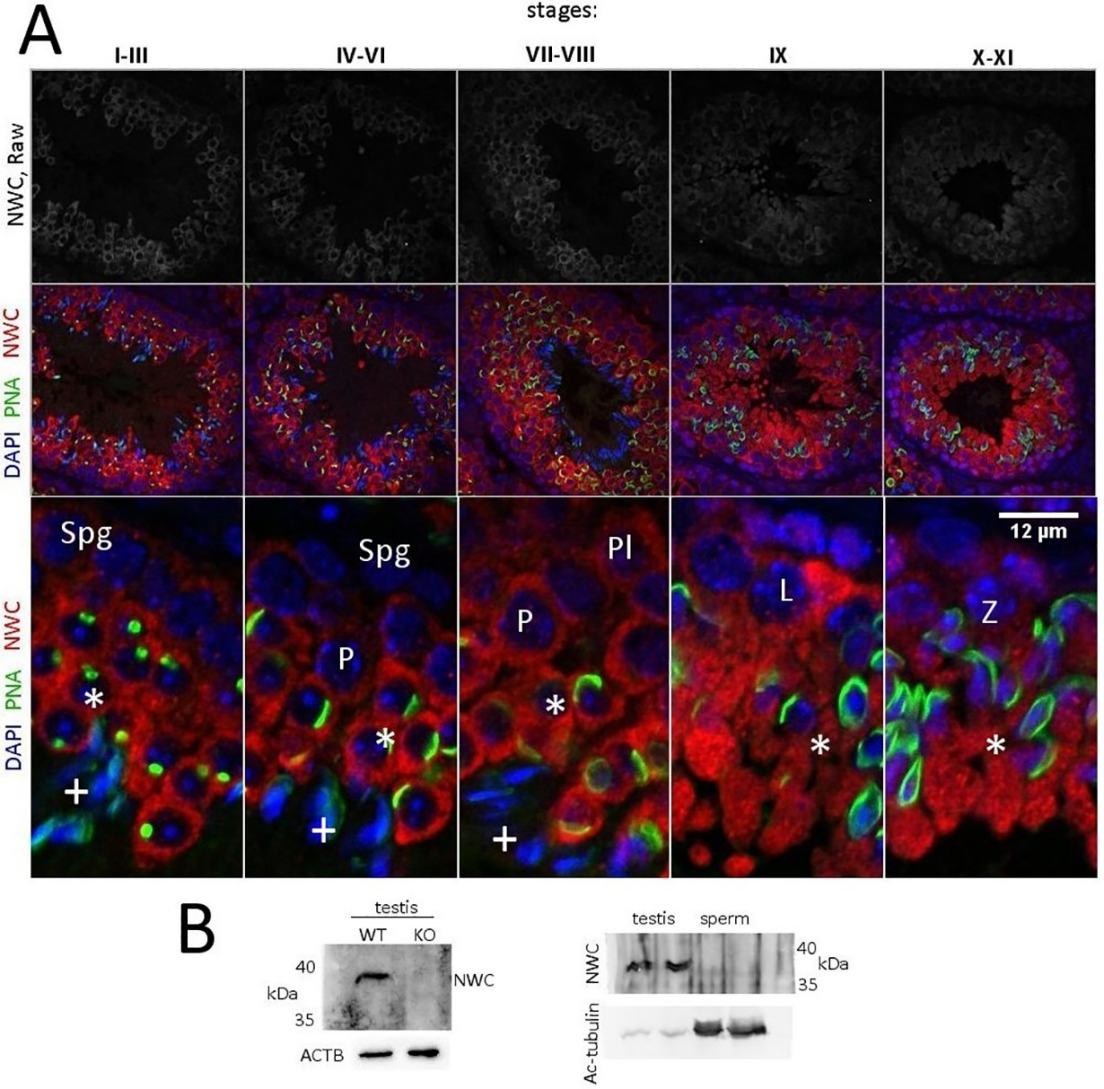
NWC expression during spermatogenesis. (A) NWC immunofluorescence in the seminiferous epithelial cycles of sexually mature WT mouse. NWC distribution is shown in red, DAPI in blue and PNA in green. Roman numbers indicate seminiferous tubule stages, Spg–spermatogonia, Pl–pre-leptotene, L–leptotene, Z–zygotene, P–pachytene, *–spermatids from steps 1 through step 11, +–Spermatids from steps 13 through 16. (B) Left: Immunoblotting NWC-KO and WT testis lysates. Each lane contains 20 μg of protein. ACTB: β-actin. Right: Immunoblotting of WT and NWC-KO epididymal sperm lysates. Each lane contains 10 μg of protein.

To determine the expression pattern of the NWC protein during spermatogenesis, we employed immunostaining of paraffin-embedded testis sections using polyclonal anti-NWC antibody and confocal microscopy. Western blot of testis lysates yielded a single band of 37 kDa molecular weight, which is not present in testis lysates obtained from an NWC-KO male (Fig 1B, left). In order to localise NWC expression during spermatogenesis, sections of seminiferous tubules were co-stained with an acrosome marker, peanut agglutinin (PNA) and classified under five stages based on Meistrich and Hess [17]. As shown in Fig 1, the NWC signal was weak or undetectable in the spermatogonia (Spg in stages I–III and stages IV–VI). NWC signal was found in preleptotene (stages VII–VIII, indicated by Pl), leptotene (stage IX, indicated by L), zygotene (stages X–XI, indicated by Z) and pachytene (stage IV to VIII, indicated by P) spermatocytes. We also immunolocalised NWC in round spermatids from step 1 to step 11 (indicated by asterisk in stages I–XI). The spermatids from steps 13 through 16 displayed no NWC signal (indicated by ‘+’ in stages I–VIII). Immunoreactive sites of NWC-containing cells were localised mainly in the cytoplasm with no clearly distinguished subcellular structures. We did not detect the NWC signal in the epididymal sperm in western blot analysis, which indicated that NWC was not directly engaged in sperm function (Fig 1B, right).

### Impaired fertilization potency of NWC-KO males in the competitive context

**Fig 2:**
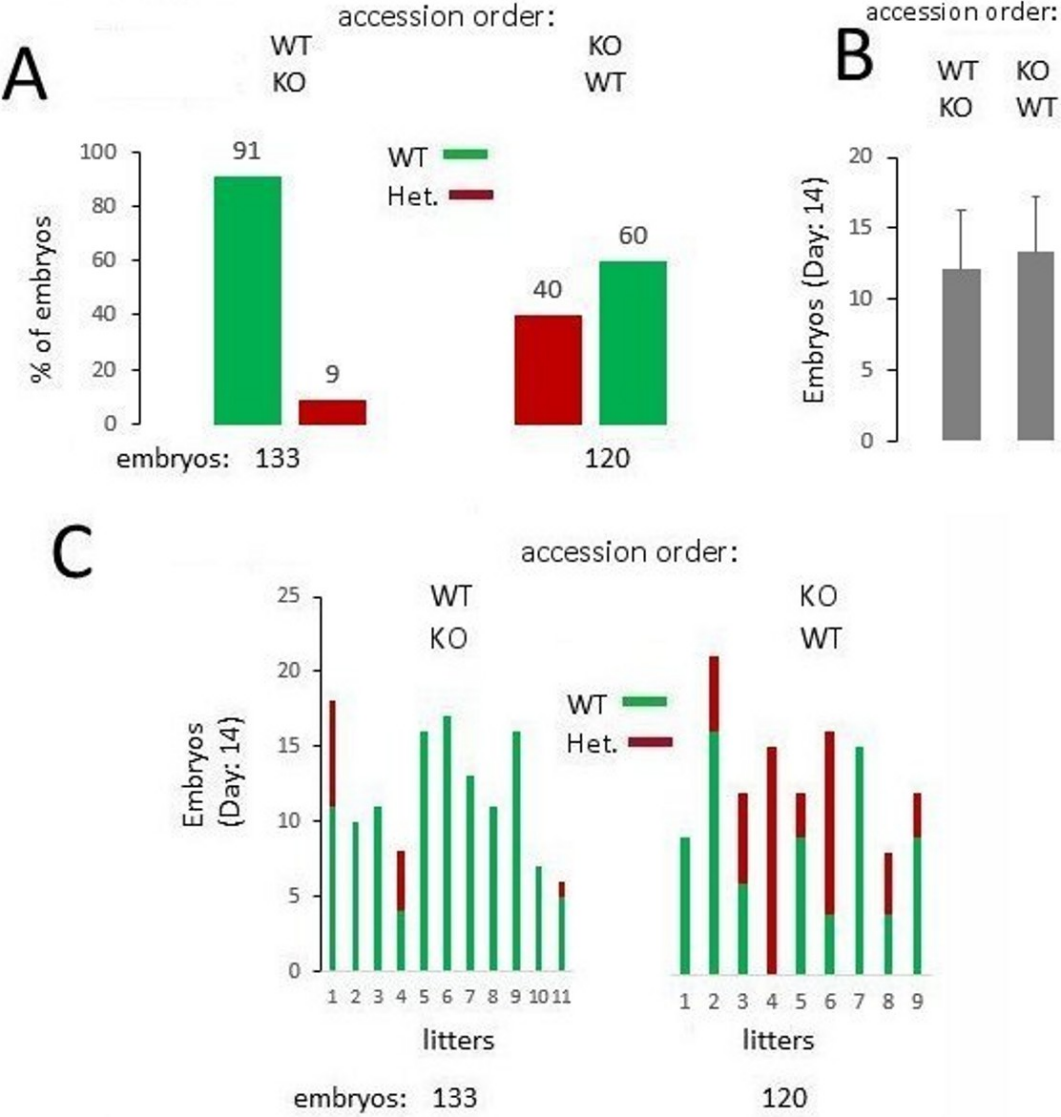
Comparison of WT and NWC-KO males fertilization potency using a sequential mating protocol mimicking polyandry and introducing sperm competition. (A) Percentage of WT and heterozygous embryos sired by WT or NWC-KO males, respectively, in two orders of access of males to the WT female. (B) Litter sizes in both access orders. Mean values with SD are shown. (C) Distribution of embryo genotypes across litters in different access orders. Each bar indicates a single litter that was genotyped on the 14^th^ day of pregnancy. Note that the presence of mixed genotypes (i.e. WT and heterozygotes) in some litters, as indicated by the bars red-and-green bars, reveals that the copulatory plug does not prevent subsequent mating.

As previously described [15], using standard mating protocols we found no difference between WT and NWC-KO mice with regard to male and female fertility as assessed by the litter size. However, in the natural environment, females mate with multiple males, which leads to sperm competition. Therefore, for a detailed characterisation of the functioning of the NWC-KO male reproductive system, we employed a more sophisticated mating protocol, i.e. sequential mating that mimics polyandry and introduces sperm competition. The protocol was based on a previously published method [7]. A single, superovulated WT female was placed in a male cage for two sequential two-hour time windows. We employed two access orders: (1) the female was first put in a WT male cage and then in a KO male cage; (2) the female was first put in a KO male cage and then in a WT male cage. This approach allowed us to reveal differences in fertilization potency between WT and NWC-KO males. When WT males had access to females during the first time window they sired 91% (out of 133) of embryos whereas NWC-KO males produced only 9% of the offspring. When the accession order was reversed, NWC-KO males were unable to reproduce the fertilisation success of WT males, siring only 40% of 120 embryos (Fig 2A–summary and Fig 2C–individual litters). The litter sizes did not change regardless of the order of access (Fig 2B).

The experiment revealed a reduced competitiveness of NWC-KO sperm. Subsequently, we attempted to identify which stages of spermatogenesis and fertilization are affected by NWC depletion. We examined the following parameters indicative of male fertility: testis weight, number of sperm recovered from epididymis and parameters concerning sperm motility and sperm morphology. Using confocal microscopy, we also examined the structure of testis sections stained with both acrosome (PNA) and manchette (α-tubulin) markers. Furthermore, we examined whether NWC depletion affected two functional capacities of the sperm: progress of capacitation and the ability of sperm to undergo acrosome reaction. Both parameters were shown to influence sperm competitiveness, as they play a critical role in fertilization [6].

### Weight of testes, sperm count, motility and morphology; visualisation of the forming acrosome and manchette structure

**Fig 3:**
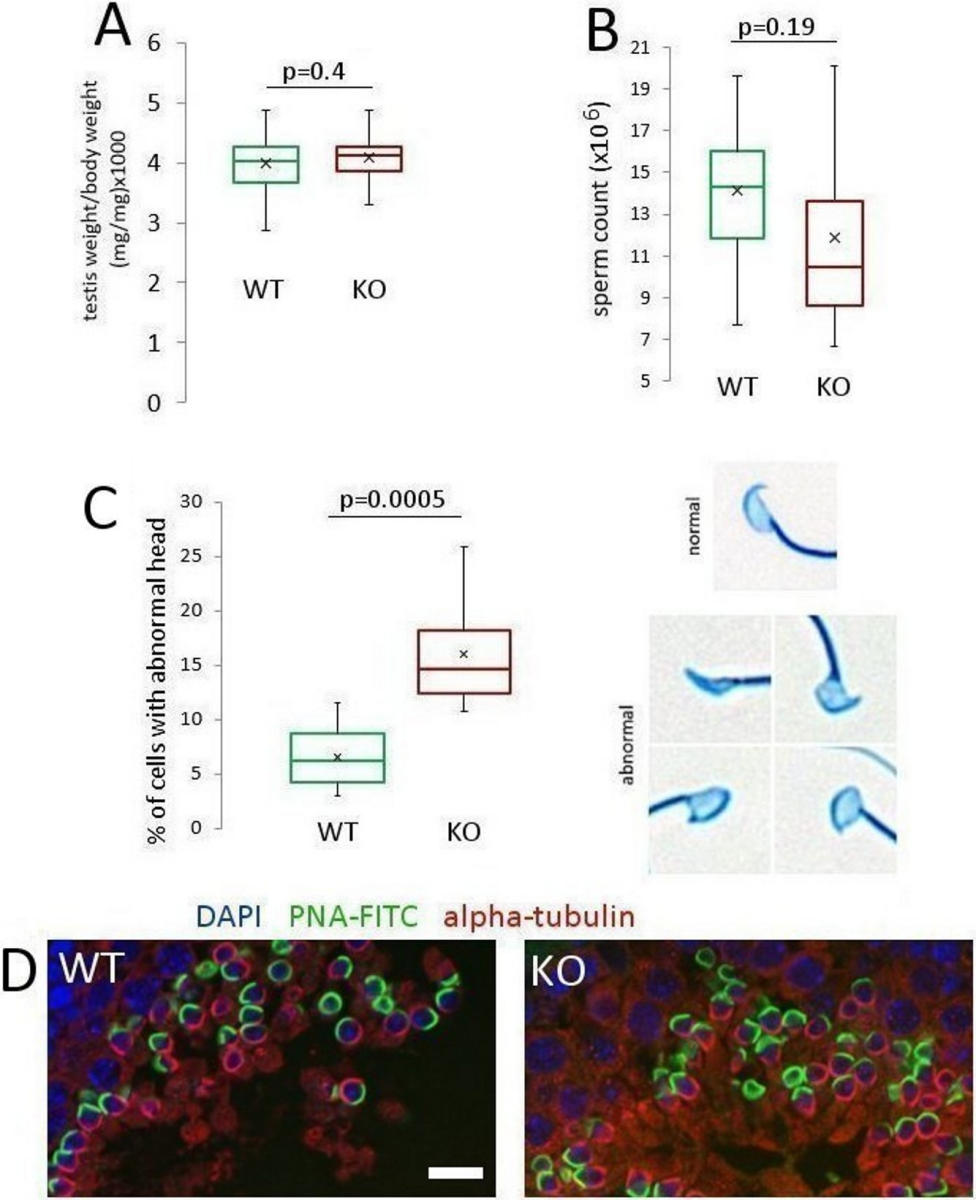
Comparison of WT and NWC-KO males in terms of selected characteristics of the male reproductive system. Analysis and comparison of (A) testis weight (WT *n* = 30 mice, NWC-KO *n* = 32 mice), (B) sperm count (WT *n* = 15 mice, NWC-KO *n* = 15 mice), (C) sperm morphology (WT *n* = 10 mice, NWC-KO *n* = 8 mice; at least 100 cells were counted for each mouse). Green and red bars represent WT and NWC-KO males, respectively. (D) Representative images of IX stage of WT and NWC-KO testis sections stained for PNA-FITC (acrosome marker; green) and α-tubulin (manchette marker; red). Scale bar– 15 μm (in both images).

WT and NWC-KO males showed a similar testis weight (Fig 3A); however, the number of sperm recovered from cauda epididymis was slightly, but not significantly, lower in the NWC-KO mice (mean values:14,1 x 10^6^ of sperm/mouse for control and 11,9 x 10^6^ of sperm/mouse for NWC-KO mice, *p* = 0.19; Fig 3B). Sperm motility analysis revealed no significant differences between both mouse types (S1 Fig). Likewise, the percentage of motile and progressive sperm was not significantly different between KO and WT mice (motile: 97.1% and 96.3%, respectively, *p* = 0.59; progressive 97.1% and 96.1%, respectively, *p* = 0.57). Analysis of Coomassie brilliant blue stained NWC-KO sperm revealed a normal structure with typical crescent-shaped acrosomes. Sperm flagella length was similar in both groups (mean values for WT = 120.5 μm, SD = 7.7 and NWC-KO = 119 μm, SD = 9.7, *p* = 0.54); however, we found an increased number of sperm with abnormally shaped heads in samples recovered from NWC-KO mice (mean values: 6.5% for control and 15.9% for NWC KO mice, *p* = 0.0005; Fig 3C). Although the number of sperm with abnormal head doubled upon *NWC* deletion, the increase in the absolute number was relatively small, and it seems unlikely that it had a significant effect on the competitiveness of NWC-KO sperm. On the other hand, this phenotypic effect indicates that NWC may be engaged in head shaping. Confocal microscopy revealed no gross differences in the structures of the manchette (as shown by alpha-tubulin staining of paraffin sections) and the forming acrosome (visualised by PNA staining) (Fig 3D). This result indicates that NWC apparently does not exert its role in sperm head shaping by influencing the structure of the manchette.

### Capacitation and acrosome reaction

**Fig 4:**
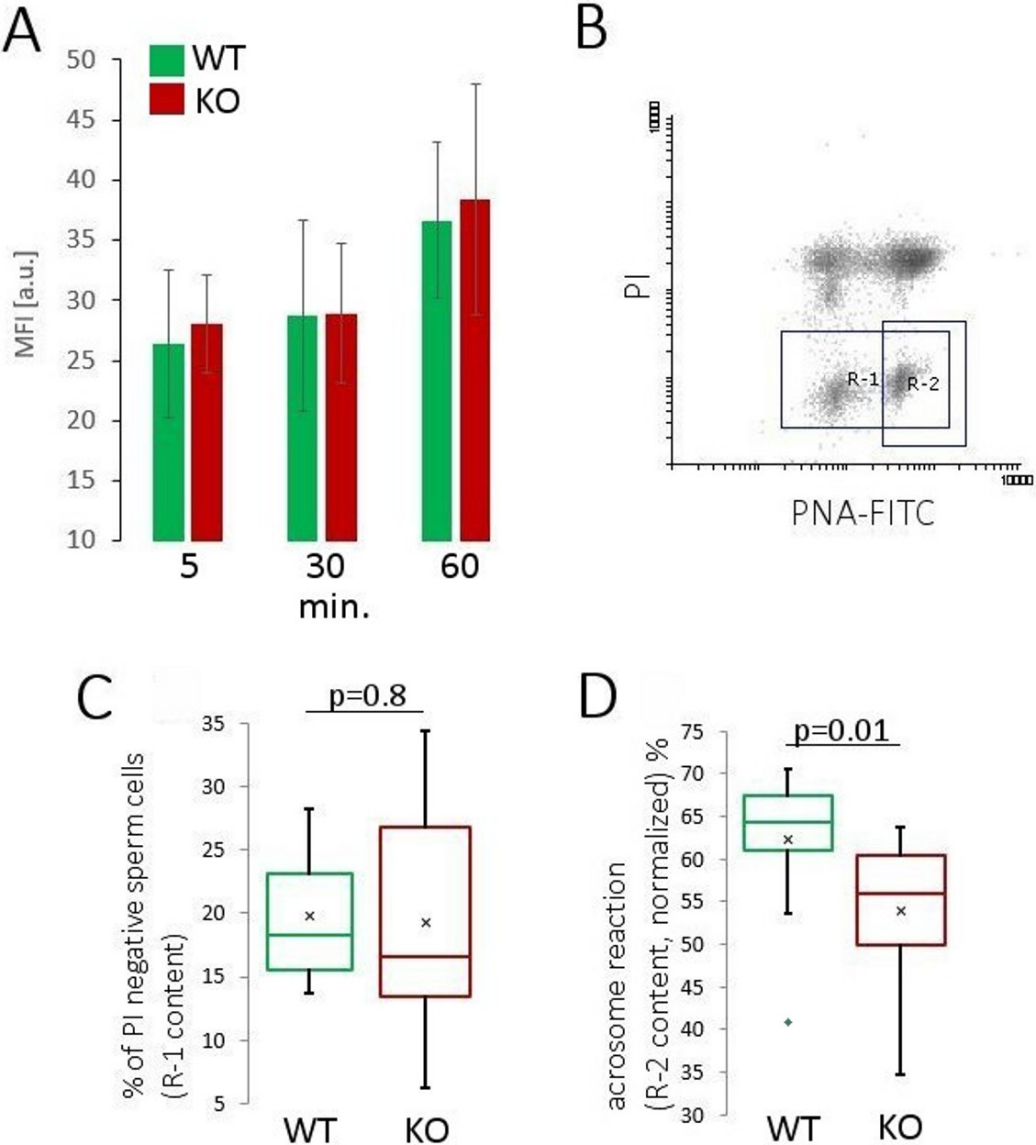
Analysis of sperm capacitation progress and response to a compound triggering acrosome reaction (calcium ionophore A23187). (A) Capacitation progress analysed by the flow cytometry of sperm recovered from WT (green bars) and NWC-KO (red bars) mice stained with anti-phospho-tyrosine antibody (anti-pY antibody). Capacitation progress was analysed at three time points (5, 30 and 60 min) in live sperm, as discriminated by fixable viability stain. Data are presented as mean values with SD from a single, representative experiment (WT, *n* = 4 mice, NWC-KO *n* = 5 mice). (B) Representative dot-plot showing flow cytometry analysis of WT sperm stained with PI and PNA-FITC after triggering acrosome reaction. The dot-plot shows four distinct populations: acrosome reacted and acrosome intact (right and left side), and PI positive and PI negative populations (upper and lower side). Two gates are shown: R-1 (PI-negative population) and R-2 (PI negative and acrosome reacted). (C) Sizes of populations of PI negative sperm. (D) Acrosome reaction analysis after the normalization of R-1 content of a given mouse to 100%. (C) and (D) present mean values obtained from *n* = 11 WT (green bars) mice and *n* = 12 NWC-KO mice (red bars).

To analyse capacitation, we employed a fluorescently labelled anti-phospho-tyrosine antibody, live/dead discrimination staining and flow cytometry. Phospho-tyrosine content was determined only in live sperm. We did not find any differences in the progress of capacitation, as indicated by phospho-tyrosine content between analysed mouse types (Fig 4A). Flow cytometry allowed for the analysis of the total phospho-tyrosine content in a particular sperm population, i.e. living cells at the time of fixation. To exclude the possibility of a lack of or diminished phosphorylation of only a single/particular protein resulting in a negligible effect detected by flow cytometry, we performed WB analysis of sperm lysates. We found no changes in tyrosine phosphorylation (S2 Fig). These results showed that sperm recovered from NWC-KO males had the same ability to capacitate as the control sperm throughout the experiment.

In the next step, we analysed acrosome reaction (AR) that was triggered after 1 h of sperm incubation in capacitating conditions by stimulating with calcium ionophore A23187. Functional status of sperm was evaluated by PNA-FITC staining and flow cytometry. The addition of propidium iodide to the samples allowed for the discrimination of non-viable (PI-positive) cells. Flow cytometry analysis of stained sperm yielded four distinct populations presented in scatter plots (PNA-FITC vs. PI) (Fig 4B). Because live sperm were stained with PNA-FITC, we considered labelled cells as acrosome reacted. We did not observe any significant differences in sperm viability after incubating the sperm in acrosome reaction-triggering conditions (Fig 4C); however, this parameter revealed a high spread around the mean value. Therefore, in the next step of the analysis, we normalised the result by treating the entire PI-negative population (gate R1 in Fig 4B) in a given mouse as 100%. Next, we determined the size of acrosome reacted (R2) and acrosome intact (R1 minus R2) populations among PI-negative sperm and found that the number of acrosome reacted cells in NWC-KO sperm was lower than in WT sperm (mean values: 62% for control sperm and 54% for NWC-KO sperm, *p* = 0.01, Fig 4D). This results indicate that sperm recovered from NWC-KO males had a diminished ability to respond to calcium ionophore. Because calcium ionophore bypasses many intracellular signalling stages, the interpretation of this finding requires knowing of the precise molecular mechanism; nonetheless, it indicates that the response to AR-inducing factor(s) may play a role in the reduced competitiveness of NWC-KO sperm.

### Assessment of cumulus mass dispersal and the efficiency of *in vitro* fertilization

Although *NWC* deletion affected sperm morphology and their ability to respond to calcium ionophore, both effects were relatively small and, therefore, unlikely to explain the reduced competitiveness of NWC-KO sperm. Therefore, in the subsequent step, we analysed the ability of the sperm of NWC-KO mice to disperse the cumulus cells layer and to fertilize eggs *in vitro*.

**Fig 5:**
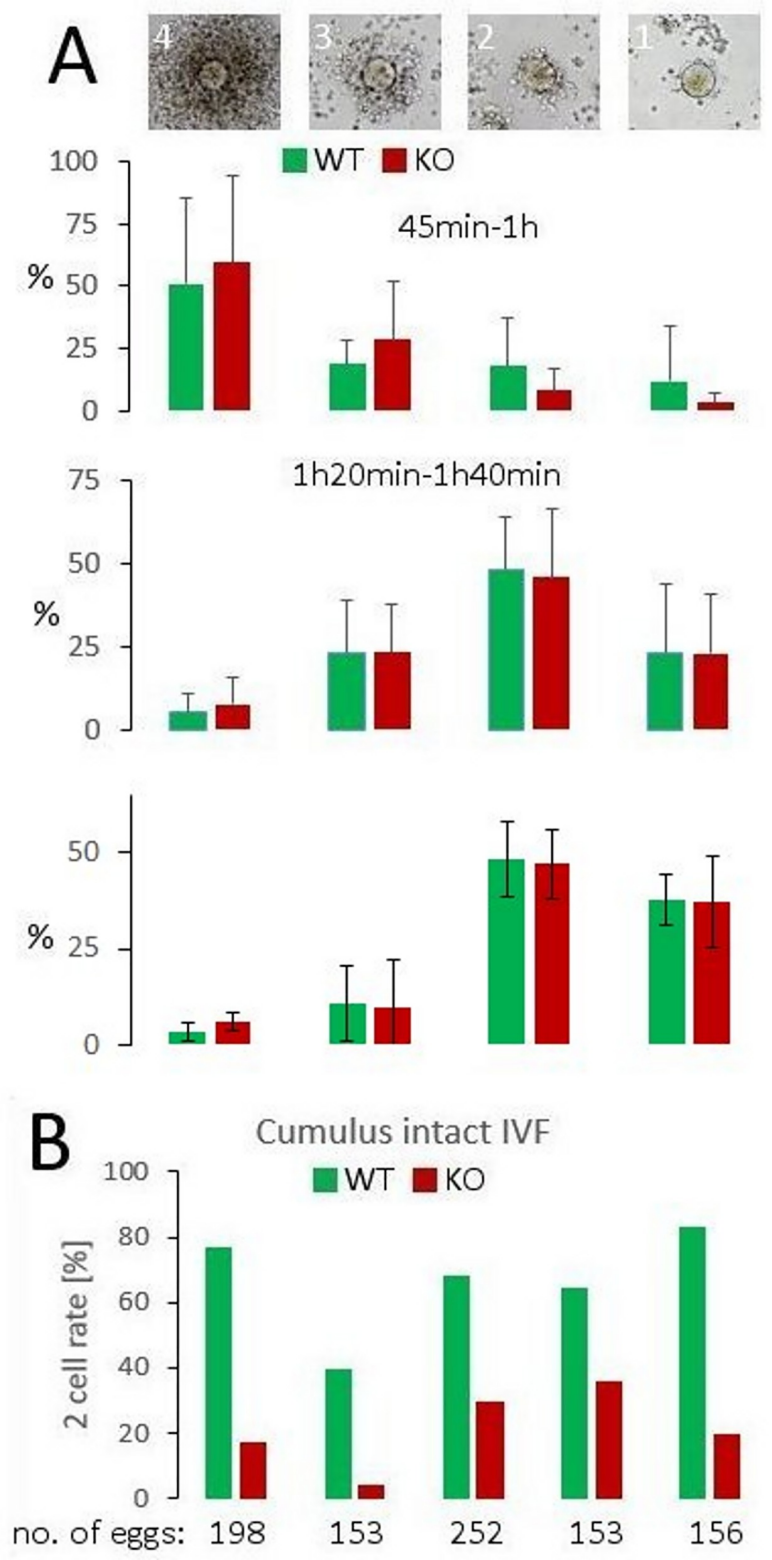
Assessment of cumulus mass dispersal and the efficiency of *in vitro* fertilization. (A) Upper part: classification key used to assess the dispersion of cumulus cells surrounding eggs. If an egg was tightly packed with cumulus cells, it was classified into group 4. Eggs were classified into group 3 when they were clearly visible but were still associated with many layers of cumulus cells, albeit not as many as in group 4. In the eggs classified into group 2, between one and two layers of cumulus cells fully surrounding the cells were retained, albeit a few layers were present at some positions. Group 1 contained eggs with only a few attached cumulus cells. Lower part: the ability of WT and NWC-KO sperm to disperse cumulus cells at the indicated time periods. The set of bars located below a given image represents the percentage of a group. Data are presented as mean values with SD obtained from at least four independent experiments. In a single experiment, a pair of males of each genotype was used as sperm donors. (B) Ability of WT and NWC-KO sperm to fertilize cumulus-intact eggs. Data are presented as the results of five independent experiments. In a single experiment, a pair of males of each genotype was used as sperm donors.

An equal number of sperm of WT and NWC-KO males were incubated with egg cells possessing an intact cumulus layer and imaged at three time periods. Eggs surrounded with masses of cumulus cells of different size were classified into four groups according to the criteria shown in the upper part of Fig 5A. Cumulus cells surrounding eggs incubated without sperm were mostly undispersed throughout the course of the experiment (more that 90% were classified into group 4 after 3h of incubation). We found that the sperm of both WT and NWC-KO males had a similar ability to disperse the layers of cumulus cells. This result indicates that the diminished fertilization potency of NWC-KO males is likely not a result of a decreased ability of the sperm to penetrate the cumulus cells (Fig 5A).

After imaging performed to assess the dispersion of cumulus cells, eggs incubated with sperm were left for 24 h. Next, the number of unfertilized (single-cell) and fertilized eggs at the two-cell stage was counted. We found that sperm recovered from NWC-KO males had a reduced ability to fertilize cumulus-intact eggs *in vitro* (Fig 5B).

## Discussion

Infertility affects approximately 5% of couples in the developed world, and the male factor contributes to about 30% of all cases [18]. Male fertility is affected by several factors, among which genetic defects are of great importance. Therefore, it is important to evaluate the role of particular genes in spermatogenesis and male fertility.

This study describes the functional phenotype of *NWC* knockout mice in terms of the male reproductive system. NWC deficiency results in: (1) reduced competitiveness, (2) increase in the number of sperm with abnormally shaped heads, (3) reduced ability of sperm to respond to an AR-triggering factor and, finally, (4) reduced ability to fertilize cumulus-intact eggs *in vitro*. The most profound effect concerns the reduced IVF rate. We postulate that it reflects the major defect of NWC-KO sperm responsible for failing the competition with WT sperm *in vivo*. However, all observed effects of *NWC* knockout possibly contribute to the reduced fertility of NWC-KO males in competitive context.

An important question raised by our study concerns the mechanism of NWC action in spermatogenesis. To gain insight into the cellular role of NWC, we determined its expression pattern during spermatogenesis. Immunolocalisation results revealed that NWC is present in the cells in those stages of spermatogenesis that encompass meiosis, as well as the acrosome formation and the first stages of manchette formation; however NWC was not localized in any particular subcellular structure. This result suggests that NWC is unlikely to be directly engaged in acrosome or manchette formation.

Previously, we identified several proteins co-immunoprecipitaing with NWC from murine testis. Interestingly, most of those proteins are involved in intraflagellar transport (IFT) [15]. Therefore, it is possible that NWC mediates its cellular role through an interaction with one or more of those proteins. Because NWC knockout have no effect on flagella length or on parameters describing sperm motility *in vitro*, we suggest that NWC is unlikely to be engaged either directly or indirectly in sperm flagella formation. One of the effects of NWC knockout is an increased number of sperm with an abnormal head. Although the observed effect is not profound, it may shed light on the role of NWC in spermatogenesis. The shape of the sperm head is a result of an interplay of three processes: nucleus elongation/condensation, development of the acrosome and the appearance of the manchette [19]. In the testis, the molecular components of the IFT system are shared with the tissue-specific intramanchette transport (IMT) system, which sorts cargo to two destinations: elongating and condensing the spermatid nucleus and developing the sperm flagella. The manchette is defined as a transient structure surrounding the nucleus, located caudally to the acrosome and containing F-actin and microtubules, which serve as tracts for molecular motor proteins [19, 20]. Proper manchette function is crucial for vesicle trafficking and, ultimately, the distribution of biologically active molecules in sperm [21]. Although we have not observed any apparent changes in the manchette structure, it is still possible that through an interaction with its molecular partner(s), NWC plays a role in the proper distribution of biologically active molecules during spermatogenesis.

One should keep in mind that the NWC protein is not present in epididymal sperm. Identifying a sperm defect produced during spermatogenesis devoid of NWC is another challenge that may approximate the role of NWC in spermatogenesis. We observed a slightly reduced population of NWC-KO sperm undergoing calcium ionophore-triggered AR. This finding suggest the engagement of NWC in establishing a signal transduction pathway leading to triggering AR, and we postulate that it occurs through the distribution or directing to the degradation of bioactive molecule(s), rather than through participation in the forming/maintaining of the acrosome and/or manchette structure.

The decreased ability of NWC-KO sperm to undergo AR may be the reason for a significant reduction of the ability to fertilize the eggs *in vitro*. Egg fertilization is a complex process involving penetration through the cumulus cells layer. Subsequent steps encompass penetration through the zona pellucida and the fusion of the sperm head with the egg. NWC depletion does not seem to affect penetration through the cumulus cells; however, we postulate that at least one subsequent step is affected by NWC depletion, because sperm from NWC-KO males fails to fertilize eggs *in vitro* as efficiently as control sperm. The normal fertility of NWC-KO males indicates that the sperm impairment is not severe enough to affect fertility, at least in standard laboratory conditions. The competitive context, on the other hand, provides a basis for phenotype demonstration, and the reduced IVF rate for NWC-KO sperm possibly indicates which fertilization stage(s) are affected by NWC depletion.

To summarise, we show that NWC-devoid spermatogenesis produces functionally impaired sperm. Based on the results presented in this paper, we suggest that NWC is engaged in the distribution of bioactive molecules, and that the functional impairment of sperm is due to defects in mechanisms triggering AR. Our work also supports the thesis that some fertility defects can only be detected in more complex conditions than those commonly used to screen for genes that are crucial for male fertility.

## Materials and methods

### Mice

The generation of NWC knock-out mice was described in a previous study, in which these mice were named NWC-KOMPcre [15]. However, in the present study, we refer to these mice as NWC-KO instead of NWC-KOMPcre for the sake of clarity. All experiments and methods were reviewed and approved by the First Local Ethical Commission for Animal Experimentation in Wroclaw at the Institute of Immunology and Experimental Therapy (Polish Academy of Sciences). Experiments on mice were performed in accordance with the regulations and guidelines provided by the First Local Ethical Commission for Animal Experimentation in Wroclaw. Animals were housed at 23°C under a 12/12 h light-dark cycle with *ad libitum* access to food and water. All experiments were performed on the group of WT (C57BL/6) and NWC-KO mice which were sexually mature (2–7 month old). The mice were euthanised by cervical dislocation performed under anaesthesia induced by isoflurane inhalation (3–5%)

### Antibodies and other reagents

Generation of rabbit anti-NWC antibody was described previously [15]. Other antibodies were purchased as follow: anti-β-actin IgG horseradish peroxidase conjugated, both anti-mouse-IgG and anti-rabbit-IgG horseradish peroxidase conjugated, mouse anti-acetylated alpha-tubulin (Santa Cruz Biotechnology); mouse anti-tubulin β3 (eBioscience); goat anti-mouse Cy5 conjugated and donkey anti-rabbit Cy2 conjugated (Jackson ImmunoResearch), rabbit anti-α-tubulin, goat anti-rabbit AF647 conjugated (Themo Fisher Scientific), anti-phospho-tyrosine antibody (pY20) conjugated with PerCP-eFluor710 (for flow cytometry) and unconjugated (for Western blot) (eBioscience). FITC conjugated Peanut agglutinin (PNA-FITC), bovine serum albumin (BSA), mineral oil, calcium ionophore A23187, propidium iodide (PI) were obtained from Sigma-Aldrich. Roti Mount medium was obtained from Carl Roth. Pregnant mare serum gonadotropin (PMSG) and human chorionic gonadotropin (hCG) were purchased from Sigma-Aldrich or Proscpec. Human tubal fluid (HTF) was prepared in the in-house Media Core Facility according to [22]. A day before experiments HTF was supplemented with BSA (0.4%), filtered through 0.22 um syringe filter, covered with mineral oil and left in the incubator (37°C and 5% CO_2_) for gas equilibration. Live-or-Dye Fixable Viability Stain was purchased from Biotium.

### Immunofluorescence and imaging

Excised testes were fixed overnight with 4% paraformaldehyde and paraffin-embedded after tissue dehydratation. The paraffin sections were cut at 4 μm thickness using microtome. Next, the sections were rehydrated, incubated in citrate buffer (pH 6.0) at 92°C for 30 min and blocked with 1% fetal bovine serum in PBS. Next, the sections were incubated with primary antibodies (antibodies were diluted in PBS; dilutions: anti-NWC 1:100; anti tubulin β3 1:100; anti α-tubulin 1:100) and PNA-FITC (1 μg/ml) for 1h. Following washing with PBS, the sections were stained with appropriate secondary antibodies: anti-mouse Cy5 conjugated, anti-rabbit Cy2 and anti-rabbit AF647 for 1h. Finally, the sections were mounted in the Roti Mount medium containing DAPI. Imaging was carried out using a Zeiss Cell Observer SD spinning disc confocal microscope equipped with a Yokogawa CSU-X1A 5000 unit (through 40x oil-immersed Plan-Apochromat objective, NA 1.3). Images were processed with ImageJ [23].

### Western blotting

Testes were mechanically disrupted using a glass homogeniser and lysed on ice in the RIPA buffer (50 mM Tris, pH 8.0, 150 mM NaCl, 0.5% sodium deoxycholate, 1% Triton X-100, 0.1% SDS) for 5 min. Insoluble fragments were removed by centrifugation (15,000 x g, 10 min, 4°C). To determine NWC presence in sperm, freshly recovered sperm (immediately after tissue discarding—see ‘Sperm recovery and counting’) were lysed on ice in RIPA buffer. For analysis of increase in phosphotyrosine content in sperm induced by capacitation 3x10^6^ of sperm were lysed at three time points (10, 60, 120 min of capacitation, see ‘Sperm capacitation’) in RIPA buffer. Insoluble fragments were removed by centrifugation (15,000 x g, 10 min, 4°C). BCA kit (Thermo Fisher Scientific) was employed to determine protein concentration. Samples were reduced and denaturated in 2x Laemmli buffer (20% glycerol, 10% 2-mercaptoethanol, 4% SDS, 0.004% bromophenol blue, 0.125M Tris HCl, pH 6.8) for 7 min at 95°C. Lysates were separated on 10% polyacrylamide gels and electro-transferred to a PVDF membrane (Millipore) in a buffer containing 25 mM Tris, 192 mM glycine and 10% methanol. After blocking in 10% skimmed milk (1 h), the membranes were incubated with a primary antibody for 1 h (dilutions: anti-NWC 1:500, anti-beta-actin horseradish peroxidase conjugated 1:1000; anti-acetylated tubulin 1:1000, anti-phosphotyrosine 1:1000). After washing, the membranes were incubated with either anti-mouse or anti-rabbit horseradish peroxidase conjugated antibodies (1 h; secondary antibodies were diluted 1:10000). The membranes were developed using enhanced chemiluminescence, and the signals were visualized with an Image Station 4000MM Pro (CareStream).

### Sequential mating

Mice were synchronised in ovulation by an intraperitoneal injection with 5 units of PMSG, followed 48h later by 5 units of hCG. A single, superovulated WT female was placed in a male cage for two sequential two-hour time windows. We employed two access orders: (1) female was first put in a WT male cage, left for 2 h, and after checking for the presence of a copulatory plug, the female was put in a NWC-KO male cage for the next 2 h and the copulatory plug was examined; (2) female was first put in a NWC-KO male cage, left for 2 h and after checking for the presence of a copulatory plug, the female was put in a WT male cage for the next 2 h and the copulatory plug was examined. The first time window in which the female is ready for insemination by a male opened 7 h after the hCG injection (at 8 p.m.), whereas the second time window opened 9 h after the hCG injection (at 10 p.m.). Following access during the first time window, the first copulatory plug was marked with a black pen. The presence of an unmarked copulatory plug detected after access during the second time window indicated second mating. To produce double-plugged females in the first access order (a WT male, then an NWC-KO male), we employed 6 WT and 7 NWC-KO males, whereas in the second access order (an NWC-KO male, then a WT male), we employed 7 WT and 7 NWC-KO males. Fourteen days after mating, the females that mated with both males as evidenced by the copulatory plug examinations, were euthanised and the embryos were genotyped by the PCR reaction, as described previously [15].

### Sperm recovery and counting

The day before experiment HTF medium was placed into the incubator for gas and temperature equilibration (37°C, 5% CO_2_). Cauda epididymis excised from WT and NWC-KO mice were placed in 0.5 ml of HTF, gently incised and left for 10 min in the incubator (37°C and 5% CO_2_) to allow sperm to swim out. After that time the tissue was discarded, the sperm suspension in HTF was covered with mineral oil and incubated for 60 min in 37°C and 5% CO_2_ for capacitation. Sperm for counting were sampled (sample volume 100 μl) immediately after tissue discard. The sperm sample was fixed in 900 μl of 4% PFA in PBS and counted in Bürker chamber.

### Computer-assisted sperm analysis

Sperm motility was analysed as described previously [24]. Briefly, cauda epididymides were dissected in a universal *in vitro* fertilization medium (Medi-Cult, Denmark). Spermatozoa were allowed to swim out of the epididymides for 5 min at 37°C and incubated for 1.5 h at 37°C, 5% CO_2_. Aliquots (13 μl) of the sperm suspension were transferred into a disposable counting chamber, which was set at a temperature of 37°C. Sperm movement was quantified using a computer-assisted semen analysis system (CEROS version 10; Hamilton Thorne Research, Beverly, MA). Approximately 2000 spermatozoa were analysed using the following parameters: average path velocity (VAP), straight line velocity (VSL), curved line velocity (VCL), lateral head amplitude (ALH), beat cross-frequency (BCF) and straightness (STR). Because all parameters were not normally distributed (even after a logarithmic or angular transformation), the nonparametric alternative for the *t* test, the Mann-Whitney *U* test, was used. For calculation, the sperm motility measurements of each parameter were pooled for each mouse type. All statistical analyses were performed using the Statistica software package (StatSoft, Inc.).

### Sperm morphology

To assess sperm morphology, 50 μl of sperm suspension in HTF, obtained immediately after recovery form cauda epididymis, were fixed by diluting in 50 μl of 4% PFA in PBS. Following 10 min of incubation, the cells were washed in 100 mM ammonium acetate (pH 8.8), spread onto a glass slide, air-dried and stained with Coomassie brilliant blue as described in [25]. Slides were photographed in the transmission light mode with a microscope (Olympus), and the images were analysed using the ‘cell counter’ plugin for the ImageJ software. Sample preparation and sperm morphology analysis were performed in a blind experiment.

### Sperm capacitation

During the 60 min incubation for sperm capacitation, samples of 100 μl were collected at three time points (5, 30 and 60 min) to determine capacitation progress. Immediately after collection, the samples were added to 500 μl of ice-cold PBS containing 1.5 μl of Live-or-Dye stain, which discriminates live and dead cells and preserves the staining pattern after fixation. After 20 min of incubation on ice, sperm were centrifuged (5 min, 500 x g) and immediately fixed in 4% paraformaldehyde for 15 min. Afterwards, the sperm were washed two times in PBS. Then sperm were suspended in 100 μl PBS with 0.1% Triton X-100 containing 0.05 μg of anti-phospho-tyrosine antibody (pY20) conjugated with PerCP-eFluor710. After 20 min of incubation, the cells were centrifuged, suspended in PBS and analysed using flow cytometry (BD FACSCalibur). PerCP-eFluor710 fluorescence was excited by a 488 nm laser line, and a 670 nm Long Pass filter was used, for detection (the FL3 channel in the device). Live-or-Dye stain was excited by a 633 nm laser line, and a 661/16 Band Pass filter was used to detect fluorescence (the FL4 channel in the device).

### Acrosome reaction

Acrosome reaction was measured based on a previously published method [26], which was modified for the purposes of the present study. Briefly, after 60 min of incubation in HTF, the sperm suspension was diluted two times with HTF. Acrosome reaction was induced by adding calcium ionophore A23187 to the samples (1.1 μl; final concentration: 1.4 μM) whereas equivalent amount of the solvent (1.1 μl of absolute ethanol) was added to the control sample. The sperm were incubated for 45 min in the presence of A23187. Afterwards, 100 μl of sperm suspension were diluted in 5 volumes of the PBS buffer, and 1 μg of PNA-FITC and PI were added to each sample. After 10 min of incubation, the samples were centrifuged (5 min, 500 x g) and suspended in PBS. Flow cytometry (BD FACSCalibur) was used to assess acrosome reaction and determine the fraction of viable and non-viable sperm cells.

### *In vitro* fertilization and assessment of cumulus cells dispersal

Females, 4–5 weeks old, were superovulated as described in previous section. Between twelve to thirteen hours after the hCG injection, metaphase II-arrested eggs surrounded with tightly packed cumulus cells were collected from the ampulla of the oviduct and placed in 40 μl of HTF (equilibrated overnight with CO_2_) covered with mineral oil. The sperm were collected from the cauda epididymis of 3–5-month-old mice and capacitated for 1h as described in previous section. The capacitated sperm (4.5 x 10^5^) of each genotype were added to a drop containing eggs. To assess cumulus cells dispersal, images were taken at the indicated time periods (Fig 5) and divided into 4 classes in a blinded evaluation. After the imaging, mixtures of eggs with sperm were left in the incubator (37°C, 5%CO_2_) for 24 h. Afterwards, fertilized (two-cell stage) and unfertilized eggs (single-cell stage) were counted.

### Statistical analysis

Data represent the mean, median and result distribution grouped into quarters. Calculations were performed using the Microsoft Excel software with the RealStats plugin (except for sperm motility parameters analysis). In all cases comparisons were made by Mann-Whitney Test (two-tailed) for two independent samples. Differences were considered significant at *p*<0.05.

## Supporting information

S1 Fig Computer-assisted sperm analysis (CASA). Sperm motility analysis by CASA revealed no significant differences between WT and NWC-KO mice. Spermatozoa were analyzed using the following parameters: average path velocity (VAP), straight line velocity (VSL), curved line velocity (VCL), lateral head amplitude (ALH), beat cross-frequency (BCF), and straightness (STR). Data obtained from WT *n* = 10 mice and NWC-KO *n* = 9 mice.
(PDF)

S2 Fig Western blot analysis of increase in phosphotyrosine (pY) content in sperm induced by capacitation. Each lane contains 10 μg of protein lysates obtained from sperm after capacitation for 10, 60 or 120 minutes. Ac-tub: acetylated alpha-tubulin.
(PDF)

S1 File The ARRIVE file. (PDF)

S2 File Supplementary data. (A) Uncropped blots that were used to make Fig 1B. (B) Raw data used to create plots presented in Fig 3A, 3B and 3C. (C) Raw data used to create plots presented in Fig 4A, 4C and 4D. (D) Raw data used to create plots presented in Fig 5A. (E) Raw data used to create plots presented in S1 Fig. (F) Raw data used to calculate the percentage of motile and progressive populations of sperm.
(PDF)
