# Supplementary material for: Lack of NWC protein (c11orf74 homolog) in murine spermatogenesis results in reduced sperm competitiveness and impaired ability to fertilize egg cells in vitro
Source: PLoS One. 2018 Dec 6;13(12):e0208649. doi: 10.1371/journal.pone.0208649 (PMC6283527; doi:10.1371/journal.pone.0208649)
Supplement: S1 Fig — Sperm motility analysis by CASA revealed no significant differences between WT and NWC-KO mice. Spermatozoa were analyzed using the following parameters: average path velocity (VAP), straight line velocity (VSL), curved line velocity (VCL), lateral head amplitude (ALH), beat cross-frequency (BCF), and straightness (STR). Data obtained from WT n = 10 mice and NWC-KO n = 9 mice. (PDF) [file pone.0208649.s001.pdf]

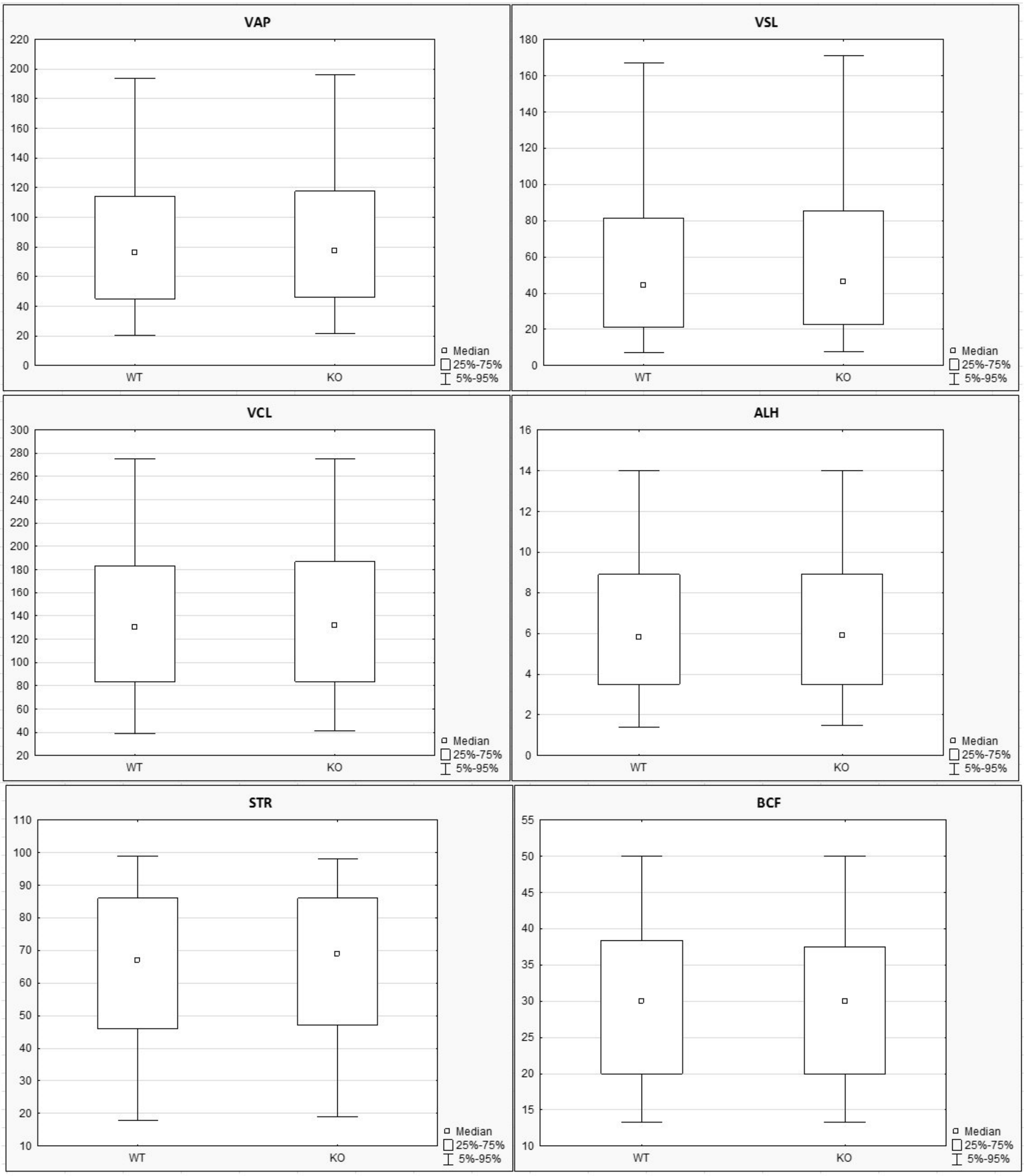

S1 Fig. Computer-assisted sperm analysis (CASA). Sperm motility analysis by CASA revealed no significant differences between WT and NWC-KO mice. Spermatozoa were analyzed using the following parameters: average path velocity (VAP), straight line velocity (VSL), curved line velocity (VCL), lateral head amplitude (ALH), beat cross-frequency (BCF), and straightness (STR). Data obtained from WT *n*=10 mice and NWC-KO *n*=9 mice
