# Supplementary material for: Lack of NWC protein (c11orf74 homolog) in murine spermatogenesis results in reduced sperm competitiveness and impaired ability to fertilize egg cells in vitro
Source: PLoS One. 2018 Dec 6;13(12):e0208649. doi: 10.1371/journal.pone.0208649 (PMC6283527; doi:10.1371/journal.pone.0208649)
Supplement: S2 Fig — Each lane contains 10 μg of protein lysates obtained from sperm after capacitation for 10, 60 or 120 minutes. Ac-tub: acetylated alpha-tubulin. (PDF) [file pone.0208649.s002.pdf]

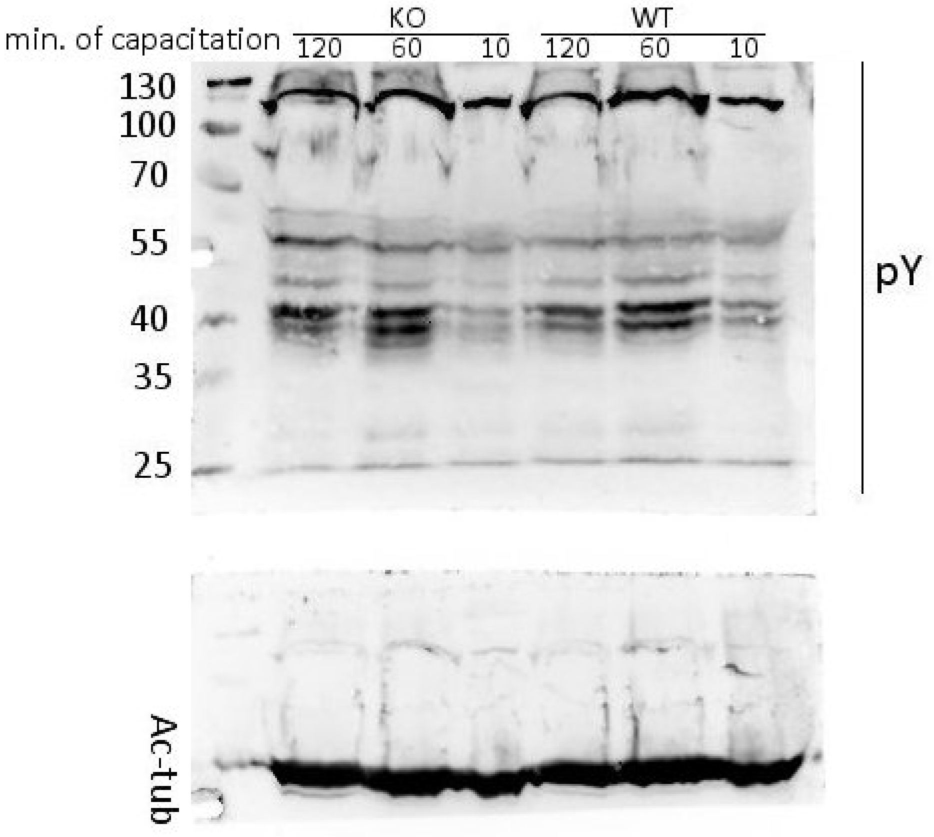

S2 Fig. Western blot analysis of increase in phosphotyrosine (pY) content in sperm induced by capacitation. Each lane contains 10 μg of protein lysates obtained from sperm after capacitation for 10, 60 or 120 minutes. Ac-tub: acetylated alpha-tubulin.
