# Supplementary material for: Lack of NWC protein (c11orf74 homolog) in murine spermatogenesis results in reduced sperm competitiveness and impaired ability to fertilize egg cells in vitro
Source: PLoS One. 2018 Dec 6;13(12):e0208649. doi: 10.1371/journal.pone.0208649 (PMC6283527; doi:10.1371/journal.pone.0208649)
Supplement: S2 File — (A) Uncropped blots that were used to make Fig 1B. (B) Raw data used to create plots presented in Fig 3A, 3B and 3C. (C) Raw data used to create plots presented in Fig 4A, 4C and 4D. (D) Raw data used to create plots presented in Fig 5A. (E) Raw data used to create plots presented in S1 Fig. (F) Raw data used to calculate the percentage of motile and progressive populations of sperm. (PDF) [file pone.0208649.s004.pdf]

S2 File. Supplementary data.

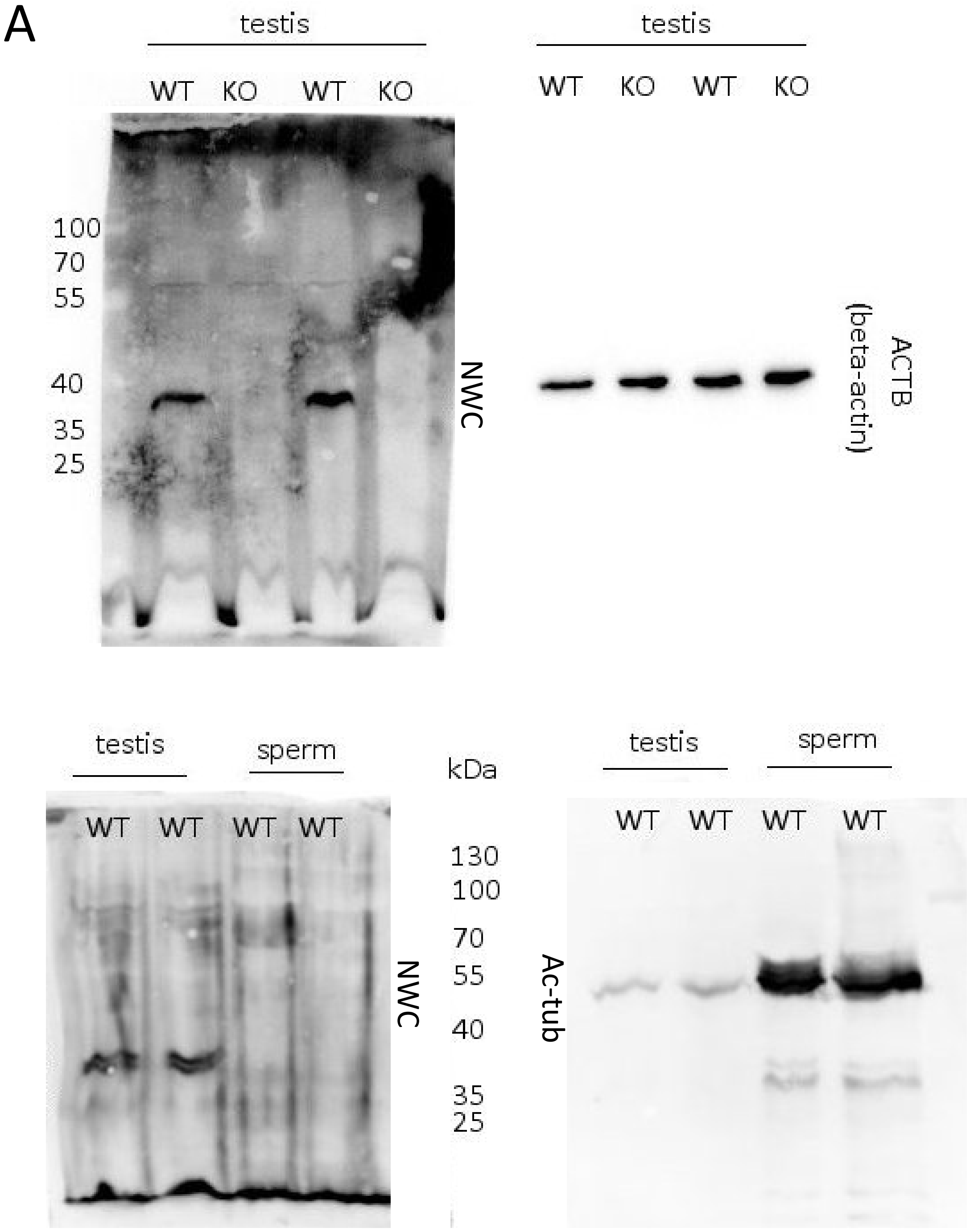

(A) Uncropped blots that were used to make Fig 1B.

B

	Fig 3A	testis weight			Fig 3B sperm count				Fig.3C	abnormal head		
	WT		KO			WT	KO		WT		KO	
	2,880658		4,615385			14,8	22			3		11,9
	3,802141		3,865376			8,3	11,2			9,43		10,8
	3,494424		4,179331			14,3	13,7			11,6		12,57
	4,732879		4,480722			12,46	10,6			4,4		17,55
	4,151684		4,40613			19,6	13,7			4		13,4
	3,651591		4,041096			14,5	8,8			4,2		19,9
	4,087838		4,089479			13,6	7,56			6,25		25,9
	4,283812		3,98291			18,8	9,4			9,8		15,88
	3,84083		4,06015			8,5	7,7			6,25	**mean**	**15,9875**
	3,844096		3,437005			12	17			6,4	**SD**	**4,71713**
	4,22591		4,110152			17	8,72		**mean**	**6,533**		
	2,880658		3,846154			11,65	6,82		**SD**	**2,716781**		
	3,617571		4,333906			7,66	8,82					
	3,568773		4,179331			24	13					
	4,195052		4,341785			15	20,1					
	3,759398		4,214559		mean	**14,14467**	**11,94133**	**x10^6**				
	3,685092		4,075342		SD	**4,472581**	**4,651**					
	3,277027		4,22929									
	4,451208		4,297922									
	3,49481		4,24812									
	3,905235		3,595377									
	4,06793		4,110152									
	4,834711		3,661538									
	4,752066		3,292308									
	4,18251		3,993174									
	4,86692		3,822526									
	4,156863		3,679245									
	4		3,396226									
	4,318182		4,223827									
	4,318182		4,187726									
**mean**	**3,977602**		4,8583									
**SD**	**0,506941**		4,453441									
		**mean**	**4,072125**									
		**SD**	**0,35277**									

(B) Raw data used to create plots presented in Figs 3A, B, C.

C

Fig 4A	capacitation								Fig 4C	% of PI negative				Fig 4D	acrosome reaction		
	t0																
KO		23,96		WT		19,715				KO		WT			KO		WT
		23,26				23,27				30,65		25,58			61		68
		30,775				28,64				22,52		27,86			56,66		67,92
		32,13				33,84				26,11		28,31			61,9		61,94
		29,89			**mean**	**26,36625**				27,3		20,69			51,43		70,51
	**mean**	**28,003**			**SD**	**6,187427**				34		18,18			55,3		64,36
	**SD**	**4,096303**								18,1		15,83			60,47		53,58
										11,74		18,95			57,14		66,63
										14,46		18,32			43,98		66,82
	t1									13,51		15,24			63,89		41,46
	702	21,885				21,6				14,3		14,46			34,68		59,98
	707	24,1				23,85				5,87		13,7			46,24		63,82
	746	30,1				30,015				9,2	**mean**	**19,73818**			55,46	**mean**	**62,27455**
	759	35,3				39,4			**mean**	**18,98**	**SD**	**5,285448**		**mean**	**54,0125**	**SD**	**8,325027**
	708	33,15			**mean**	**28,71625**			**SD**	**8,981529**				**SD**	**8,593461**		
	**mean**	**28,907**			**SD**	**7,961379**											
	**SD**	**5,760045**															
	t2																
	702	29,725				29,375											
	707	27,7				34,98											
	746	41,51				37,04											
	759	50,6				45,03											
	708	42,35			**mean**	**36,60625**											
	**mean**	**38,377**			**SD**	**6,482884**											
	**SD**																

(C) Raw data used to create plots presented in Figs 4A, C, D.

D

**Fig 5A**															
45min-1h min															
classes	WT				mean	SD			KO				mean	SD	
4	55,4	2,4	84	62	**50,95**	**34,59918**			45	17,5	93	83	**59,625**	**34,87448**	
3	24,3	7,1	16	28	**18,85**	**9,302509**			33,3	59,4	7	15,3	**28,75**	**23,19547**	
2	17,56	45	0	10	**18,14**	**19,29694**			16,6	14,8	0	1,5	**8,225**	**8,684229**	
1	2,7	45	0	0	**11,925**	**22,0867**			5	8,1	0	0	**3,275**	**3,987794**	
1h20min	1h40min														
classes	WT				mean	SD			KO				mean	SD	
4	6,9	11,8	0	4	**5,675**	**4,967478**			19,1	2,8	1,6	7,4	**7,725**	**7,984725**	
3	34,5	35,3	21,3	1,3	**23,1**	**15,88794**			43	8,5	19	22,3	**23,2**	**14,45199**	
2	51,7	29,4	68,1	42,6	**47,95**	**16,25638**			29,8	54,3	71,4	28	**45,875**	**20,82008**	
1	6,9	23,5	10,6	52	**23,25**	**20,4448**			7,46	34,3	8	41,8	**22,89**	**17,77239**	
2h-2h20min															
classes	WT					mean	SD		KO					mean	SD
4	2,2	5,7	0	5,5	2,7	**3,22**	**2,399375**		4,4	6,8	4,8	4,1	9,4	**5,9**	**2,222611**
3	6,7	26	0	13	6,9	**10,52**	**9,799847**		4,4	31	2,4	2,04	9,4	**9,848**	**12,18325**
2	53,3	40,9	63	39	46	**48,44**	**9,842916**		57	43,8	47,6	53	33,8	**47,04**	**8,955892**
1	38	27,2	37	42,6	44,4	**37,84**	**6,701343**		34	17,8	45,2	40,8	47,3	**37,02**	**11,88915**

(D) Raw data used to create plots presented in Fig 5A.

E

GENOTYPE	ANIMAL NUMBER	VAP	VAP	VAP	VAP	VAP	VAP	VSL	VSL	VSL	VSL	VSL	VSL	VCL	VCL	VCL	VCL	VCL	VCL
		mean	valid	SD	median	percentile 5	percentile 25	mean	valid	SD	median	percentile 5	percentile 25	mean	valid	SD	median	percentile 5	percentile 25
KO	507	99,24875	1959	59,74974	88,60000	25,80000	55,40000	72,51588	1959	58,88166	56,20000	10,10000	29,70000	153,4057	1959	75,69135	147,0000	49,20000	94,0000
KO	508	76,95367	1932	55,25917	64,65000	17,60000	34,75000	54,10999	1932	52,70497	37,20000	6,10000	16,20000	122,6133	1932	71,31671	111,6000	33,00000	66,2000
KO	509	95,62574	2172	57,16953	85,45000	26,00000	52,40000	67,01142	2172	54,90369	52,50000	9,00000	26,20000	150,6740	2172	74,33472	143,9000	49,20000	93,0000
KO	510	89,62487	2155	56,72246	77,50000	22,00000	47,40000	61,70770	2155	54,04267	45,90000	8,30000	23,00000	141,1204	2155	74,48418	131,0000	41,40000	83,7000
KO	511	88,59692	1946	59,92394	76,00000	21,20000	45,60000	60,57071	1946	57,06102	43,60000	7,30000	21,00000	140,7918	1946	77,80652	129,7000	38,50000	81,1000
WT	526	85,02270	1978	51,00299	76,35000	24,50000	47,80000	59,10212	1978	50,09848	46,65000	7,70000	23,40000	137,8033	1978	66,33302	131,2000	47,80000	87,1000
WT	546	97,13797	2310	59,01686	86,80000	27,00000	54,40000	68,93403	2310	57,75201	54,30000	9,60000	27,10000	155,1883	2310	74,69868	146,3500	55,00000	101,1000
WT	580	98,26867	2017	59,75832	90,00000	22,50000	54,20000	68,26564	2017	59,16962	53,10000	7,80000	24,20000	152,5628	2017	76,17860	147,1000	42,20000	93,8000
WT	584	89,97504	1703	59,59993	77,40000	21,10000	46,60000	63,16383	1703	56,95347	47,30000	8,50000	23,60000	139,8070	1703	79,34409	126,9000	37,50000	82,0000
KO	588	93,13864	2047	53,42237	81,90000	29,40000	54,50000	67,14211	2047	52,51482	52,40000	11,20000	29,60000	148,0223	2047	71,05843	140,1000	51,50000	92,8000
KO	589	75,13149	1864	51,83239	63,65000	17,50000	37,45000	51,01078	1864	48,20829	36,90000	6,90000	17,00000	120,6053	1864	69,07749	109,2000	32,30000	68,1000
KO	592	88,93046	2088	55,43970	79,75000	20,90000	47,30000	61,70235	2088	53,85449	45,20000	7,20000	21,60000	143,6169	2088	72,80094	134,2000	43,10000	87,5000
KO	593	91,30601	1980	58,52295	79,20000	24,00000	48,15000	62,58869	1980	56,42623	45,40000	7,90000	22,25000	149,4778	1980	77,61967	140,5500	45,90000	89,7000
WT	608	73,95383	1698	56,32934	61,35000	14,30000	32,50000	49,87744	1698	51,42984	33,00000	6,10000	14,90000	116,8891	1698	74,59195	101,4500	27,90000	57,2000
WT	625	86,20201	2093	50,84353	77,90000	24,20000	48,70000	59,04510	2093	48,89559	45,00000	7,90000	23,20000	140,8731	2093	68,71951	134,9000	44,60000	90,4000
WT	626	91,93132	2018	58,09641	81,45000	25,20000	49,60000	63,94708	2018	56,97658	48,55000	8,50000	23,40000	149,1680	2018	73,88066	140,1500	48,40000	94,4000
WT	664	61,03446	682	46,14200	47,95000	13,30000	27,50000	39,84106	682	40,65131	23,20000	5,30000	11,80000	102,4611	682	68,44338	83,8500	26,30000	48,8000
WT	665	83,89116	1120	61,03881	70,20000	17,30000	40,70000	56,87732	1120	57,92143	40,00000	5,60000	16,85000	136,7656	1120	79,84717	125,0500	31,40000	78,9000
WT	666	82,25752	1975	57,05289	69,30000	19,80000	41,40000	56,73635	1975	53,81255	40,70000	7,00000	19,40000	135,0404	1975	76,66556	122,0000	38,20000	77,0000

GENOTYPE	ANIMAL NUMBER	ALH	ALH	ALH	ALH	ALH	ALH	BCF	BCF	BCF	BCF	BCF	BCF	STR	STR	STR	STR	STR	STR
		mean	valid	SD	median	percentile 5	percentile 25	mean	valid	SD	median	percentile 5	percentile 25	mean	valid	SD	median	percentile 5	percentile 25
KO	507	6,818479	1959	3,935642	6,200000	1,500000	3,700000	29,00243	988	10,59258	27,70000	13,80000	20,00000	68,65799	1959	23,84923	74,00000	22,00000	52,00000
KO	508	5,733023	1932	3,637792	5,000000	1,300000	3,000000	30,71523	1057	11,44354	30,00000	13,30000	21,80000	64,27174	1932	25,18029	68,00000	18,00000	46,00000
KO	509	6,918324	2172	4,019633	6,300000	1,700000	3,700000	30,12310	1078	10,88204	30,00000	13,30000	22,50000	65,40700	2172	24,62255	69,00000	21,00000	46,00000
KO	510	6,647425	2155	4,136531	5,900000	1,500000	3,500000	28,95115	1179	11,41715	28,60000	13,00000	20,00000	64,43666	2155	25,22280	68,00000	19,00000	46,00000
KO	511	6,569013	1946	4,069188	5,900000	1,400000	3,400000	30,61996	1062	11,12427	30,00000	13,30000	21,80000	63,19681	1946	25,57537	66,00000	17,00000	44,00000
WT	526	6,586249	1978	3,720433	6,000000	1,700000	3,800000	29,87420	1097	11,38102	28,90000	13,30000	20,00000	64,74014	1978	24,67115	68,00000	17,00000	48,00000
WT	546	7,064026	2310	3,984765	6,500000	1,800000	4,000000	30,22186	1130	11,08282	30,00000	13,30000	21,40000	66,31472	2310	24,73035	70,00000	20,00000	47,00000
WT	580	6,890977	2017	4,038041	6,100000	1,600000	3,800000	28,90366	1038	11,03190	28,10000	13,30000	20,00000	64,35845	2017	25,54037	68,00000	18,00000	46,00000
WT	584	6,532824	1703	4,095485	5,600000	1,200000	3,300000	28,87846	910	11,23436	27,70000	13,00000	20,00000	65,57369	1703	24,62344	69,00000	21,00000	47,00000
KO	588	6,789643	2047	3,964970	6,200000	1,500000	3,700000	29,20150	1067	10,44673	30,00000	13,30000	20,00000	68,40498	2047	23,89100	73,00000	21,00000	53,00000
KO	589	5,965236	1864	3,849324	5,200000	1,300000	3,000000	29,50272	1031	11,41087	30,00000	13,30000	20,00000	63,19903	1864	24,74370	66,00000	18,00000	45,00000
KO	592	6,673467	2088	3,929122	5,900000	1,600000	3,700000	29,90871	1102	11,09554	30,00000	13,80000	20,80000	64,02969	2088	25,35226	67,00000	18,00000	45,00000
KO	593	6,927273	1980	4,007158	6,400000	1,600000	3,900000	30,34626	1042	11,34265	30,00000	14,10000	21,40000	63,02879	1980	25,04092	65,00000	19,00000	45,00000
WT	608	5,695171	1698	4,080487	4,700000	1,000000	2,500000	30,25291	979	11,50528	30,00000	13,30000	20,00000	62,67138	1698	25,25930	64,00000	19,00000	43,00000
WT	625	6,513569	2093	3,763944	5,900000	1,500000	3,500000	30,99811	1111	11,36397	30,00000	13,80000	22,50000	64,06355	2093	24,98438	68,00000	19,00000	45,00000
WT	626	6,860406	2018	3,910519	6,200000	1,700000	3,900000	30,78174	1035	10,83166	30,00000	14,40000	22,20000	64,32656	2018	24,88396	67,00000	18,00000	46,00000
WT	664	5,164076	682	3,747788	4,300000	1,000000	2,100000	31,06842	399	11,73410	30,00000	13,30000	21,40000	60,95161	682	26,13091	65,00000	15,00000	40,00000
WT	665	6,346786	1120	4,078995	5,600000	1,200000	3,300000	30,92583	569	11,57100	30,00000	13,80000	21,80000	62,10357	1120	26,33530	65,00000	16,00000	42,00000
WT	666	6,379089	1975	3,968648	5,600000	1,400000	3,300000	29,44440	1063	11,19706	28,80000	13,30000	20,00000	63,80506	1975	25,20266	67,00000	18,00000	46,00000

(E) Raw data used to create plots presented in S1 Fig

F

motile (%)	progressive (%)	animal number	genotype
98	98	593	KO
98	98	592	KO
89	88	664	WT
98	98	666	WT
95	95	665	WT
93	92	608	WT
98	98	626	WT
98	98	625	WT
97	97	580	WT
98	98	584	WT
99	99	509	KO
99	99	588	KO
92	92	589	KO
98	98	526	WT
98	98	510	KO
97	97	511	KO
95	95	508	KO
98	98	507	KO
99	99	546	WT

(F) Raw data used to calculate the percentage of motile and progressive populations of sperm.
